# A Japanese single-center experience of the efficacy and safety of asfotase alfa in pediatric-onset hypophosphatasia

**DOI:** 10.1186/s13023-022-02230-y

**Published:** 2022-02-23

**Authors:** Yohei Sugiyama, Taijiro Watanabe, Makiko Tajika, Tetsuro Matsuhashi, Masaru Shimura, Takuya Fushimi, Keiko Ichimoto, Ayako Matsunaga, Tomohiro Ebihara, Tomoko Tsuruoka, Tomoyuki Akiyama, Kei Murayama

**Affiliations:** 1 Center for Medical Genetics, Chiba Children’s Hospital, 579-1 Heta-cho, Midori-ku, Chiba City, Chiba Prefecture 266-0007 Japan; 2 Department of Metabolism, Chiba Children’s Hospital, Chiba, Japan; 3 Department of Neonatology, Chiba Children’s Hospital, Chiba, Japan; 4 Department of Child Neurology, Okayama University Hospital, 2-5-1 Shikata-cho, Kita-ku, Okayama city, Okayama Prefecture 700-8558 Japan

**Keywords:** Alkaline phosphatase, Asfotase alfa, Enzyme replacement therapy, Hypophosphatasia

## Abstract

**Background:**

Hypophosphatasia (HPP) is a rare inherited metabolic disorder caused by mutations in the *ALPL* gene, which encodes tissue nonspecific alkaline phosphatase. The severity of HPP is widely diverse from the perinatal form to the adult mild form. The former represents the most severe form and was earlier associated with high mortality due to pneumonia which was caused by severe hypomineralization of the bones—such as chest deformity and fractured ribs—and muscle weakness. Enzyme replacement therapy using asfotase alfa (AA) was approved in 2015 in Japan for treating patients with HPP and has improved their pulmonary function and life prognosis. There are several practical and ethical challenges related to using orphan drugs for a rare disorder in a publicly funded healthcare system. Sharing experiences about their application is essential towards formulating guidelines to assist clinicians with decisions about their initiation and withdrawal. We report the details of AA experience in ten cases of pediatric-onset HPP in nine families from January 2015 to November 2019 (median [interquartile range] age 11.0 [7.6–12.5] years; 60% male). This is a study of a single-center cohort describing the clinical course of patients with HPP, mainly consisting of the mild childhood form of HPP, treated with AA in Japan.

**Results:**

One case of perinatal form of HPP, two cases of benign prenatal form, and seven cases of childhood form were observed. The most common symptom at onset was pain. All patients had low serum alkaline phosphatase levels as compared to the age-matched reference range before the commencement of AA. All HPP patients seem to have responded to AA treatment, as evidenced by pain alleviation, increased height standard deviation, improvement in respiratory condition and 6-min walk test result improvement, disappearance of kidney calcification, alleviation of fatigue, and/or increases in bone mineralization. There were no serious adverse events, but all patients had an injection site reaction and skin changes at the injection sites. Genetic analysis showed that eight out of ten patients had compound heterozygosity.

**Conclusions:**

AA may be effective in patients with mild to severe pediatric-onset forms of HPP.

**Supplementary Information:**

The online version contains supplementary material available at 10.1186/s13023-022-02230-y.

## Background

Hypophosphatasia (HPP) is a genetic disorder caused by defects in tissue-nonspecific alkaline phosphatase (TNSALP) [1]. The loss-of-function mutations in the *ALPL* gene, located in the short arm of chromosome 1, consisting of 12 exons, and encoding TNSALP, results in HPP [2]. *ALPL* variants occur in all exons. Depletion of TNSALP causes accumulation of phosphoethanolamine (PEA), pyridoxal 5' phosphate (PLP), and inorganic pyrophosphate. TNSALP dephosphorylates PLP into pyridoxal (PL), which can cross the blood–brain barrier and subsequently become rephosphorylated to PLP. In the brain, PLP is required for the biosynthesis of many neurotransmitters [3]. The central pathogenesis of HPP is impaired bone calcification, and the mechanism by which reduced TNSALP activity leads to hypocalcification is well understood [4]. TNSALP produces inorganic phosphate by degrading pyrophosphate, a calcification inhibitor [5]. The inorganic phosphate produced is taken up into the matrix vesicles released from osteoblasts [6, 7]. In HPP, it is thought that the accumulation of pyrophosphate and the decrease in local phosphorus concentration due to the decreased activity of TNSALP causes hypocalcification [8].

Patients with HPP have varied clinical manifestations and are classified as having one of the six forms (perinatal, benign prenatal, infantile, childhood, adult, and odontohypophosphatasia) based on the age of onset and severity [1, 9, 10]. There is a Japanese clinical practice guideline to diagnose patients with pediatric and adult HPP [11]. HPP has diverse symptoms, such as seizures, poor body weight gain, and premature deciduous tooth loss due to decrement in serum alkaline phosphate (ALP) levels [12]. A low ALP level is important for the diagnosis of HPP. HPP is diagnosed based on clinical symptoms, radiological findings, and biochemical test results by clinicians who are familiar with the disease. Genetic testing is recommended for supporting diagnosis and genetic counseling [11, 13]. The incidence of severe phenotypes in Japan is approximately 1 in 150,000, whereas the incidence of other phenotypes is unclear [11]. In Japan, the common *ALPL* variants are c.1559delT and c.979T>C. The prevalence of the c.1559delT allele is approximately 1/480 [10]. Genetic carriers of HPP are usually asymptomatic, although some are mildly symptomatic. It is observed that the more severe the disease, the more often it is subject to autosomal recessive inheritance [14].

Enzyme replacement therapy using asfotase alfa (AA) has been approved in many countries, including the United States, the European Union, Canada, and Japan. A Japanese clinical trial has demonstrated the efficacy of AA in treating patients with HPP [15]. There were 13 patients in this trial: six with perinatal form, five with infantile form, one with childhood form, and one with the adult form of HPP. In Japan, AA is covered by health insurance, and the guidelines for its use as an established treatment protocol in patients with HPP facilitate its accessibility and availability to healthcare professionals and patients living with HPP as compared with these in other countries that do not provide insurance coverage or do not have regulatory approval for this treatment for adult-onset HPP [11]. There is some literature describing improvements in the clinical course of the infantile and childhood form of HPP, with rachitic change after AA treatment [16]. Nevertheless, it is necessary to share experiences from various centers to generalize the applicability of this novel pharmacotherapy for HPP; thus, we present a case series of pediatric-onset HPP and report our experience in treating patients with HPP, particularly the mild childhood form, with AA at our center.

## Methods

This is the observational retrospective patient study and did not involve any research-based patient intervention. All Japanese patients with HPP who visited Chiba Children’s Hospital, Japan between January 2015 and November 2019 were included in this study. The diagnosis of HPP was established based on the criteria laid down by the Japanese Clinical Practice Guidelines for HPP [11]. The guidelines define the primary clinical symptoms as bone mineralization disorder and premature loss of deciduous teeth, and the primary laboratory finding as low serum ALP levels compared with age-specific normal values [17]. If one or more primary symptoms are observed on clinical examination along with a low serum ALP level, HPP should be suspected, and gene testing would be performed for supporting diagnosis. This Japanese guideline is for diagnosis and states the recommendations of AA indication.

*ALPL* gene analysis was performed with informed consent. Ten children (4 girls and 6 boys) from 9 families were included. The demographic characteristics, clinical features, laboratory investigations, and genetic analysis of all patients are summarized in Table 1. The classification of the clinical form of HPP was based on age at onset, symptoms, and severity of radiological findings [18]. The serum PLP, PL, and PLP/PL level reference values were determined based on a study published by Akiyama et al. [19], and that for PEA was from LSI Medicine Corporation. The serum ALP level reference values were determined based on a study published by Tanaka et al. [17]. Short stature was defined as a height of − 2 standard deviation (SD) below the mean for the respective age as specified by the 2000 growth survey on body mass index for age references for Japanese children (cross-sectional national survey data by the Japanese Ministry of Health, Labour and Welfare, and the Ministry of Education, Culture, Sports, Science, and Technology) [20, 21]. AA treatment involved subcutaneous administration thrice weekly at a dose of 2 mg/kg or six times a week at a dose of 1 mg/kg, with the maximum volume of a single injection being 1 mL (40 or 100 mg/mL). We routinely measured the bone density using dual X-ray absorption (DXA), biochemical data, kidney echography and carpus radiography data, limb pain, and 6-min walk test (6MWT) results depending on the symptoms and development of patients.

**Table 1:** Demographic characteristics, clinical features, laboratory investigation, and genetic analysis of patients

Case		Clinical form of HPP		Sex		Age at onset of HPP		Symptom at first onset		Age at AA treatment introduction		Age at last follow-up		ALP before AA treatment (lower limit of standard value by age)		Genetic variants (NM_000478.6)	Family history of HPP		Orthopedic findings on X-ray		Renal calcification		Pain		Periodontal disease	
1		Mild childhood		M		2 years		Foot pain		5 year 0 months		7 years 0 months		197 (< 430) IU/L		c.613G>A (p.Ala205Thr)/c.1559delT (p.Leu520fs)	−		+		N.C		+		+	
2		Mild childhood		F		0 years 6 months		Poor weight gain		10 years 1 months		12 years 0 months		400 (< 470) IU/L		c.984_986delCTT (p.Phe327del) Heterozygous variant	−		+		−		+		−	
3		Mild childhood		M		1 years		Premature loss of deciduous tooth		1 years 5 months		3 years 2 months		79 (< 395) IU/L		c.526G>A (p.Ala176Thr)/c.920C>T (p.Pro307Leu)	−		+		−		−		+	
4		Mild childhood		M		9 years 10 months		Pain in knee and hip		11 years 5 months		12 years 2 months		421 (< 470) IU/L		c.1559delT (p.Leu520fs) Heterozygous variant	−		−		−		+		−	
5		Mild childhood		M		7 years		Short stature		10 years 4 months		12 years 7 months		359 (< 460) IU/L		c.382G>A (p.Val128Met)/c.529G>A (p.Ala177Thr)	+		+		−		+		−	
6		Mild childhood		F		10 years		Knee pain		17 years 1 months		18 years 7 months		135 (< 130) IU/L		c.382G>A (p.Val128Met)/c.529G>A (p.Ala177Thr)	+		+		−		+		−	
7		Mild childhood		M		4 years 2 months		Premature loss of deciduous tooth		4 years 3 months		5 years 2 months		113 (< 430) IU/L		c.572A>G (p.Glu191Gly)/c.1559delT (p.Leu520fs)	−		+		−		−		+	
8		Benign prenatal		M		At birth		Unequal length of limbs		16 years 3 months		21 years 1 months		103 (< 270) IU/L		c.979T>C (p.Phe327Leu)/c1559delT (p.Leu520fs)	−		+		+		−		+	
9		Benign prenatal		F		At birth		Bilateral femur deformity		1: 4 years 5 months 2: 6 years 3 months		9 years 4 months		157 (< 430) IU/L		c.568_570delAAC (p.Asn190del)/c.979T>C (p.Phe327Leu)	−		+		+		−		−	
10		Perinatal		F		At birth		Short limbs		At birth		2 years 4 months		8 (< 530) IU/L		c.1333T>C (p.Ser445Pro)/c.1559delT (p.Leu520fs)	−		+		N.C		−		−	

Patient 5 is the younger brother of patient 6

ALP, alkaline phosphatase; AA, asfotase alfa; HPP, hypophosphatasia; N.C., not conducted

All diagnostic procedures and treatment protocols were based on established protocols intended to diagnose and treat patients, and adhered to the principles of the Declaration of Helsinki 1975, as revised in 2000. The assay for pyridoxal phosphate and pyridoxal was approved by the Research Ethics Board at Okayama University Hospital (Approval No. 1603-012).

## Results

### Clinical data of patients with HPP

The cases discussed here consist of one case of perinatal form, two cases of benign prenatal form, and seven cases of mild childhood form of HPP.

#### Case 1: 7-year-old boy

The patient began experiencing pain in his lower legs at 2 years of age. At 4 years, he had pain in the upper arms and exhibited premature loss of three deciduous teeth. The serum ALP level was 197 IU/L (reference value for this age: 430–1200 IU/L). X-ray findings of flaring at the distal ulnar metaphyseal end and cupping at the distal metaphyseal ends of both radii were noted. Genetic testing revealed that he had compound heterozygosity for c.613G>A (p.Ala205Thr) and c.1559delT (p.Leu520fs) of the *ALPL* gene. We initiated AA treatment, and there was consequent improvement in his symptoms of pain in the lower extremities and a gradual decline in the rate of premature loss of deciduous teeth. The patient’s height increased from 104 cm (− 0.8 SD below the mean for his age group as per the 2000 growth survey) [21] to 117 cm (− 0.5 SD) 2 years after the initial presentation. His 6MWT result improved from 350 m (70% of normal) to 400 m (75% of normal) after initiation of AA treatment. Both cupping and flaring at the forearm had disappeared 2 years after the commencement of AA treatment. He experienced mild lipoatrophy at injection sites. This case is that of a mild childhood form of HPP.

#### Case 2: 12-year-old girl

The patient had poor weight gain, and her radiographs showed rachitic changes at the ends of the bones of the trunk at 6 months of age. At 1 year of age, she was suspected to have HPP as her serum ALP levels were in the range of 260–380 IU/L (reference value for this age: 395–1289 IU/L), but she had no other symptoms. Thereafter, her height was − 1.0 to − 2.5 SD below the mean for her age group as per the 2000 growth survey [21]. At 8 years of age, she experienced neck pain and lumbago. There were no abnormalities on imaging tests, and HPP was once again suspected. Genetic analysis revealed a heterozygous variant of the *ALPL* gene, c.984_986delCTT (p.Phe327del). Her father had the same variant but was asymptomatic; therefore, this variant was not thought to be the cause of her symptoms. The patient was rehabilitated and treated with anti-inflammatory drugs. At the age of 10 years, she became unable to attend school on account of her inability to walk, carry her bag by herself, or play with her friends because of pain in her extremities (Additional file 1). She was referred to our hospital to commence AA treatment as the serum PLP level was elevated at 164.4 nmol/L (reference value 14.5–57.3 nmol/L); subsequently, the pain in her extremities gradually decreased. Approximately 3–4 months after the initiation of AA treatment, she was able to join an athletic festival without the support of any anti-inflammatory drugs. Six months after AA treatment initiation, she did not have any difficulty in attending school (Additional file 1). Her 6MWT result improved from 380 m (60% of normal) to 433 m (68% of normal) after AA treatment initiation. She experienced mild lipoatrophy and mild redness at injection sites. This case is that of a mild childhood form of HPP.

#### Case 3: 3-year-old boy

The patient lost five deciduous teeth prematurely with roots intact when he was aged 1 year and 4 months and one tooth when aged 1 year and 5 months. He visited a dentist who suspected HPP. He was then referred to our hospital for further investigation and treatment. His height was 74 cm (− 2.0 SD), and radiography showed bilateral distal ulna cupping. The serum ALP level was 79 IU/L (reference value for this age: 395–1339 IU/L), and the c.526G>A (p.Ala176Thr) and c.920C>T (p.Pro307Leu) *ALPL* variants were detected. We started AA treatment at age 1 year and 5 months. He lost one tooth when he was aged 2 years and 3 months; since then, there had been no tooth loss till the last follow-up at age 3 years and 2 months. His height increased from 74 cm (− 2.0 SD) to 87 cm (− 1.8 SD) 1 year and 7 months after AA treatment initiation. Radiography showed disappearance of cupping of the distal ulna 2 years after AA treatment initiation. He experienced mild lipoatrophy with mild redness at injection sites. This case is that of a mild childhood form of HPP.

#### Case 4: 12-year-old boy

At 9 years of age, the patient complained of pain in the right knee and hip joint and was referred to an orthopedic surgeon. No abnormalities were noted on radiography, but the patient was monitored. Over the next several months, he developed pain in his chest and extremities and was referred to our hospital for a detailed investigation. Blood examination revealed a low serum ALP level of 421 IU/L (reference value for this age: 470–1500 IU/L). Genetic testing for the *ALPL* gene exhibited a heterozygous c.1559delT variant (p.Leu520fs). Based on our experience with treating case 2 of this series, that of a patient with HPP with a heterozygous variant responsive to AA treatment, we likewise commenced AA treatment in case 4 at the age of 11 years. The patient’s pain gradually subsided after the introduction of AA treatment. He experienced mild lipoatrophy with mild redness at injection sites. This case is that of a mild childhood form of HPP.

#### Case 5: 12-year-old boy

At the age of 7 years, the patient had a short stature at − 2.0 SD as per the 2000 growth survey [21]. Blood examination by a general practitioner revealed that the serum ALP level was 338 IU/L (reference value for this age: 450–1250 IU/L). He was referred to our hospital at the age of 9 years for further examination and treatment. Orthopedic examination and radiography revealed a spur on the right radial diaphysis, lumbar kyphosis, strong pelvic anteversion, and hip flexion contracture. His serum ALP level continued to remain low, and he had developed bilateral ankle pain, which prompted us to perform further examinations. The *ALPL* variants c.382G>A (p.Val128Met) and c.529G>A (p.Ala177Thr) were found, and AA treatment was initiated. His height increased from 127 cm (− 1.9 SD) to 142 cm (− 1.4 SD) in 2 years and 3 months, and his pain gradually improved after the introduction of AA treatment, although radiographic findings did not improve. He experienced lipohypertrophy and lipoatrophy with skin redness at injection sites. This case is that of a mild childhood form of HPP.

#### Case 6: 18-year-old girl

This patient, the older sister of patient 5 of this series, experienced left knee pain at the age of 10 years and was treated surgically after a diagnosis of meniscus injury, but the knee pain persisted. She was also diagnosed with scoliosis at 13 years of age. When she was 15 years old, her younger brother (case 5) was diagnosed with HPP. Consequently, she was suspected to have the same disease. Blood examination revealed a low serum ALP level of 140 IU/L (reference value for this age: 120–570 IU/L). We diagnosed her with HPP. Genetic testing showed the same *ALPL* variants as her brother, which was consistent with the diagnosis. At 16 years of age, AA treatment was initiated. Her knee pain gradually improved, and she experienced considerably less fatigue in her daily life after the initiation of AA treatment. Her 6MWT result also improved from 356 m (54% of normal) to 410 m (65% of normal) after AA treatment initiation. She experienced mild lipoatrophy at injection sites. This case is that of a mild childhood form of HPP.

#### Case 7: 5-year-old boy

At 4 years of age, the patient lost a deciduous tooth with the root intact. He was taken to a general practitioner, and his serum ALP level was found to be 116 IU/L (reference value for this age: 430–1200 IU/L). Thereafter, he was referred to our hospital for further examination and treatment. Radiography revealed band sclerosis at the distal femoral metaphyseal end. A genetic test revealed *ALPL* variants—c.572A>G (p.Glu191Gly) and c.1559delT (p.Leu520fs)—and the diagnosis of HPP. Nine months after AA treatment initiation, he had not experienced any further premature loss of deciduous teeth. Radiographs showed that band sclerosis tended to improve, with no significant change in the bone permeability 3 months after AA treatment initiation. He experienced mild redness at injection sites. This case is that of a mild childhood form of HPP.

#### Case 8: 21-year-old male

At birth, the patient had a shorter right thigh and a shorter right upper arm than the left thigh and left upper arm, respectively. He had no significant complications other than the differences in the length of his upper arms and lower legs. At the age of 2 years, his serum ALP level was 103 IU/L (reference value of this age: 410–1250 IU/L). Subsequently, a short stature at approximately − 2.0 SD as per the 2000 growth survey [21] and periodontal disease were noted. At the age of 8 years, he was referred to our hospital for surgical correction of the difference in the length of his lower legs, and the operation was performed. When the patient was 15 years old, AA was approved in Japan. He had bilateral kidney calcification at this time. Genetic testing was performed before treatment, and the *ALPL* gene variants c.979T>C (p.Phe327Leu) and c1559delT (p.Leu520fs) were found; hence, AA was commenced. Subsequently, his 6MWT results improved from 463 m (67% of normal) to 560 m (81% of normal), and kidney calcification also resolved. He experienced lipohypertrophy and lipoatrophy with skin redness at injection sites. This case is that of a benign prenatal form of HPP.

#### Case 9: 9-year-old girl

Fetal ultrasound at 29 weeks of age revealed bilateral femoral deformities. A low serum ALP level (96 IU/L; reference value for this age: 530–1610 IU/L) at birth had shown and she was diagnosed HPP. Genetic testing revealed *ALPL* variants, namely c.568_570delAAC (p.Asn190del) and c.979T>C (p.Phe327Leu). She subsequently had a short stature of less than − 2.0 SD and also developed bilateral kidney calcification. AA treatment was started at the age of 4 years for short stature, although treatment was discontinued within one week due to injection pain. When she was 6 years and 3 months old, AA treatment was resumed due to concerns regarding her short stature and kidney calcification. She experienced gradual alleviation in fatigue after the initiation of AA treatment, although mild kidney calcification persisted after 1 year and 4 months of AA treatment. Her height had increased from 103 cm (− 2.4 SD) to 119 cm (− 2.3 SD) at age 3 years and 1 month. She experienced lipohypertrophy and lipoatrophy with skin redness at injection sites. This case is that of a benign prenatal form of HPP.

#### Case 10: 2-year-old girl

The patient had prominently shorter limbs than normal since the fetal period and was born at 38 weeks and 3 days of gestation. She had severe respiratory failure immediately after birth and was therefore intubated. Blood test results after birth revealed a low serum ALP level of 8 IU/L (reference value for this age: 530–1610 IU/L). Consequently, she was clinically diagnosed as having HPP and was started on AA treatment and vitamin B6 supplements on day 0. A genetic test later revealed the *ALPL* variants c.1333T>C (p.Ser445Pro) and c.1559delT (p.Leu520fs) and the diagnosis of HPP. The patient has sensorineural hearing loss that is gradually improving. She needs respiratory support during the night, but the respirator can be taken off during the daytime. She could pull herself up at the age of 2 years. Although she still had severe short stature, her height increased from 35 cm (− 6.5 SD) to 73 cm (− 4.8 SD) in 2 years and 4 months after AA treatment initiation. She experienced mild lipoatrophy at injection sites. This case is that of a perinatal form of HPP.

To summarize, our case series reports the details of AA treatment experience in ten patients with HPP from 9 families from January 2015 to November 2019 (median [interquartile range] age 11.0 years [7.6–12.5] years; 60% male patients) (Table 2). There was one case of perinatal form, two cases of benign prenatal form, and seven cases of mild childhood form of HPP. The most common symptom at onset was pain in the limbs (three out of ten patients: cases 1, 4, and 6). The most common symptoms during the clinical course of the disease were pain (five out of ten patients: cases 1, 2, 4, 5, and 6) and short stature (five out of ten patients: cases 2, 3, 8, 9, and 10). Two patients (cases 8 and 9) had bilateral kidney calcification. All ten patients had low serum ALP levels before AA treatment as compared with the age-matched reference range.

**Table 2:** Clinical findings and laboratory values before and after asfotase alfa (AA treatment)

Case	Height [cm] (SD score)	Improvement in clinical findings	ALP [IU/L]	PLP [nmol/L]	PL [nmol/L]	PLP/PL (reference)	PEA/Cr (µmol/g Cr)	6MWT [meter (% of normal)]	Bone density [g/cm^2^] (SD score)	Adverse effects
1	104 (− 0.8 SD)/117 (− 0.5 SD)	Pain alleviation Growth in height 6MWT improvement Improvement of X-ray finding	197/12977	316.1/22.9 (16.2–57.4)	13.1/22.2 (8.8–28.0)	24.1/1(1.6–3.3)	0.14/0.11 (0.08–1.08)	350 (70%)/400 (75%)	0.463 (N.R.)/0.522 (N.R.)	+
2	125 (− 2.0 SD)/136 (− 2.0 SD)	Pain alleviation Improvement of activity intensity 6MWT improvement	400/32956	164.4/3.2 (14.5–57.3)	49.1/8.9 (7.4–17.7)	3.4/0.4 (1.2–2.5)	0.02/0.08 (0.08–1.08)	380 (60%)/433 (68%)	0.554 (− 2.8)/0.578 (− 2.7)	+
3	74 (− 2.0 SD)/87 (− 1.8 SD)	Improvement of premature teeth loss Growth in height Improvement of X-ray finding	79/24700	2293/137.1 (16.2–57.4)	39/96 (8.8–28.0)	58.7/1.4 (1.6–3.3)	0.5/0.18 (0.08–1.08)	−	0.266 (N.R.)/−	+
4	143 (− 0.3 SD)/146 (− 0.5 SD)	Pain alleviation	421/10274	100.8/− (14.5–57.3)	19.5/− (7.4–17.7)	5.2/− (1.9–5.3)	0.019/− (0.08–1.08)	330 (50%)/−	0.630 (− 2.0)/0.732 (− 1.2)	+
5	127 (− 1.9 SD)/142 (− 1.4 SD)	Pain alleviation Growth in height	359/22743	108.2/21.9 (14.5–57.3)	17.5/43.5 (7.4–17.7)	6.2/0.5 (1.9–5.3)	0.03/0.03 (0.08–1.08)	505 (80%)/523 (81%)-	0.636 (− 1.4)/0.758(− 0.8)	+
6	155 (− 0.7 SD)/155 (− 0.7 SD)	Alleviation of pain and fatigue 6MWT improvement	135/24675	−/3.0 (14.5–57.3)	−/4.1 (7.4–17.7)	−/0.7 (1.9–5.3)	0.08/− (0.08–1.08)	356 (54%)/410 (65%)	0.873 (− 1.8)/0.872 (− 1.9)	+
7	97 (− 1.2 SD)/103 (− 1.3 SD)	Cessation of premature teeth loss Improvement of X ray finding	113/4051	1938/152 (16.2–57.4)	33/72.6 (8.8–28.0)	58.7/2.1 (1.6–3.3)	0.16/0.09 (0.08–1.08)	−	0.362 (N.R.)/0.330 (N.R.)	+
8	155 (− 2.5 SD)/157 (− 2.4 SD)	6MWT improvement Disappearance of kidney calcification	103/44368	−/37.1 (N.R.)	−/185.5 (N.R.)	− /0.2 (N.R.)	0.05/0.015 (0.08–1.08)	463 (67%)/560 (81%)	0.685 (− 3.8)/0.684 (− 4.0)	+
9	103 (− 2.4 SD)/119 (− 2.3 SD)^a^	Alleviation of fatigue Growth in height	157/25634	−/15.7 (16.2–57.4)	−/34.3 (8.8–28.0)	−/0.5 (1.9–5.3)	0.12/0.09 (0.08–1.08)	300 (57%)/−	0.478 (N.R.)/0.626 (− 1.5)	+
10	35 (− 6.5 SD)/73 (− 4.8 SD)	Respiratory condition improvement Growth in height	8/17720	−/27 (N.R.)	−/37.6 (N.R.)	−/0.7 (N.R.)	−/0.29 (0.08–1.08)	−	−/0.249 (N.R.)	+

Values before the slash indicate the values before the start of AA treatment, and after the slash indicate the values after the start of AA treatment

ALP, alkaline phosphatase; AA, asfotase alfa; N.R., no reference; PEA, phosphatidylethanolamine; PL, pyridoxal, PLP, pyridoxal phosphate; 6MWT, 6-min walk test

^a^The height of patient 9 was measured at the age of 6 years 3 months before restarting AA treatment for a second time

Genetic analysis showed that eight out of ten patients had compound heterozygosity, and two patients (cases 2 and 4) had only one heterozygous variant. Five out of ten patients had the c.1559delT (p.Leu520fs) variant, which is common in Japan [10]. In contrast, only two patients had the c.979T>C (Phe310Leu) variant, which is also common in Japan [10].

All patients showed improvements in clinical symptoms after AA treatment, such as increase in height, pain alleviation, fatigue alleviation, improvement in premature deciduous tooth loss, better performance in the 6MWT, or improvement in respiratory insufficiency. All five patients (cases 1, 2, 4, 5, and 6) who experienced pain in extremities had alleviation of pain after AA treatment initiation. In one patient (case 3) with premature loss of deciduous teeth with the roots intact, the interval of losing teeth seems to have reduced after initiation of AA treatment. In case 7, the patient had not experienced further premature loss of deciduous teeth 9 months after AA treatment. Clinical improvements in patients with the benign prenatal form (cases 8 and 9) were increased distance in the 6MWT, and fatigue alleviation. Kidney calcification resolved in case 8, but not in case 9. The patient in case 10 had severe short stature, with a height of 73 cm (− 4.8 SD) at 2 years and 4 months of age even after the initiation of AA treatment (her height was − 6.5 SD at birth), although respiratory insufficiency improved, and motor, mental, and auditory development progressed gradually. Biochemical analysis after the initiation of AA treatment showed a decrease in PLP, PLP/PL, and PEA values. These changes are consistent with the pathophysiology of the disease when treated with AA. Serum PLP levels of patients in cases 2, 6, and 9 decreased to subnormal levels after AA treatment initiation.

In three patients (cases 5, 6, and 9), AA treatment was either discontinued temporarily or the total dose was reduced owing to injection site reaction or fever. Patient 5 resumed adequate dose AA treatment 6 months after dose reduction. Patient 6 continues to receive AA treatment twice a week instead of three times a week owing to pain due to AA treatment injection. Patient 9 resumed AA treatment 1 year and 10 months after discontinuation due to concerns about her short stature and kidney calcification. We did not encounter anaphylactic shock, significant fluctuations of serum calcium and phosphorus levels, or ectopic calcification due to long-term treatment with AA as far as we know. All patients experienced injection site reaction to varying degrees and some patients experienced lipohypertrophy and lipoatrophy (Additional file 2). There were no other complications that could be caused by AA.

## Discussion

This was a single-center cohort study in Japan describing the clinical course of patients with HPP treated using AA. We have shown some efficacy of AA treatment in pediatric-onset HPP without serious complications; however, there were some limitations in our study. First, effectiveness of AA in reducing pain and fatigue was assessed based on the subjective responses of the patients or their parents. Second, our study group was a single-center cohort. Nevertheless, we believed that presenting the clinical courses of patients with HPP during AA treatment would benefit clinicians treating patients with HPP.

Although the efficacy of AA in the perinatal and infantile form of HPP has been demonstrated in several countries, including Japan, the number of patients with the childhood and adult forms of mild HPP was comparatively lower [15, 22]. There is a monitoring guidance for patients with HPP treated with AA, which suggests regular monitoring of biochemistry, skeletal radiographs, respiratory function, growth, pain, mobility and motor function, and quality of life [23].In our study, not all patients had rachitic changes on radiographs, and thus these patients with the childhood form appeared to have milder findings compared to those in the clinical trial [22], wherein all patients had rachitic changes on radiographs and the childhood form was severe. In our study, clinical findings such as short height, premature loss of teeth, poor performance in the 6MWT, or respiratory insufficiency were improved after initiating AA treatment. Some patients reported improvements regarding only subjective parameters such as pain and fatigue. To quantify these symptoms, it is necessary to use dedicated questionnaires. Although there is no published report about AA treatment reducing premature loss of teeth in HPP, it seems that AA treatment reduced the interval of losing teeth in some of our patients. It has been demonstrated that DXA scans are likely not useful in HPP, though we saw improvement in bone density with DXA scans in some patients [24].

Long-term treatment of patients with mild HPP with a conventional dosage of AA might have a hypothetical risk of leading to excessively low inorganic pyrophosphate levels, which may have effects such as ectopic mineralization. A similar condition is generalized arterial calcification of infancy (GACI), which is characterized by reduced levels of PPi in plasma and can be lethal in infancy. In GACI, it has been postulated that reduced PPi is a major determinant for ectopic mineralization, since PPi is a powerful mineralization inhibitor [25]. In Japan, AA dosage can be adjustable at the discretion of the attending physician, although there is no guideline or evidence about dosage adjustment. In cases 2, 6, and 9, we considered reducing the dosage of AA since their serum PLP level dropped to a subnormal level.

Case 10 with perinatal HPP continued to have severe short stature even though the growth rate improved considerably after the initiation of AA treatment (from − 6.5 to − 4.8 SD). We could not draw a comparison between the clinical signs before and after the initiation of AA treatment with respect to case 10 as AA treatment was initiated immediately after birth. The natural prognosis of perinatal HPP is poor, with death generally occurring within 1 year [26]. Therefore, we can say that AA treatment improved the patient’s prognosis.

Although genetic analysis is recommended for diagnosing HPP, a detailed patient history and clinical examination are the crucial first steps. There might be patients with heterozygous *ALPL* variants, like in case 2, who respond to AA treatment. We should comprehensively consider the indications for starting AA treatment in patients with HPP and closely monitor their clinical improvement while evaluating the safety and complication of long-term AA treatment for each patient. Further studies are needed to explore the improvements in clinical signs and to assess the safety and complications associated with various forms of HPP after AA treatment.

## Conclusions

We reported the single-center cohort in Japan describing the clinical course of patients with HPP, treated with AA. Every patient seemed to benefit from the AA treatment without serious adverse effects. We will continue to conduct future studies with long-term follow-up periods to investigate the efficacy and safety of AA treatment in patients with HPP.

## Supplementary Information

**Additional file 1.** Video record of patient 2 demonstrating the patient’s inability to walk and the subsequent improvement in pain and walking ability after asfotase alfa (AA) initiation. Description of data: At the age of 10 years, the patient could not attend school on account of her inability to walk, carry her bag by herself, or play with her friends because of pain. The pain in her extremities gradually improved after the introduction of AA treatment. Three to four months after AA treatment initiation, she could join an athletic festival without the support of any anti-inflammatory drugs. Six months after AA treatment initiation, she did not have any difficulty attending school.

**Additional file 2**. Skin changes noted in each case.

**Additional file 3.** Japanese prevalence of the each variant with Japanese Multi Omics Reference Panel.

## Data Availability

The datasets used and/or analyzed during the current study are available from the corresponding author on reasonable request.

## Abbreviations

AA: Asfotase alfa
DXA: Dual X-ray absorption
HPP: Hypophosphatasia
PEA: Phosphoethanolamine
PLP: Pyridoxal 5′ phosphate
SD: Standard deviation
TNSALP: Tissue-nonspecific alkaline phosphatase
6MWT: 6-Minute walk test
