# Supplementary material for: A Japanese single-center experience of the efficacy and safety of asfotase alfa in pediatric-onset hypophosphatasia
Source: Orphanet J Rare Dis. 2022 Feb 23;17:78. doi: 10.1186/s13023-022-02230-y (PMC8867653; doi:10.1186/s13023-022-02230-y)
Supplement: Supplementary file 1 — Additional file 1. Video record of patient 2 demonstrating the patient’s inability to walk and the subsequent improvement in pain and walking ability after asfotase alfa (AA) initiation. Description of data: At the age of 10 years, the patient could not attend school on account of her inability to walk, carry her bag by herself, or play with her friends because of pain. The pain in her extremities gradually improved after the introduction of AA treatment. Three to four months after AA treatment initiation, she could join an athletic festival without the support of any anti-inflammatory drugs. Six months after AA treatment initiation, she did not have any difficulty attending school. [file 13023_2022_2230_MOESM1_ESM.pptx]

## Slide 1
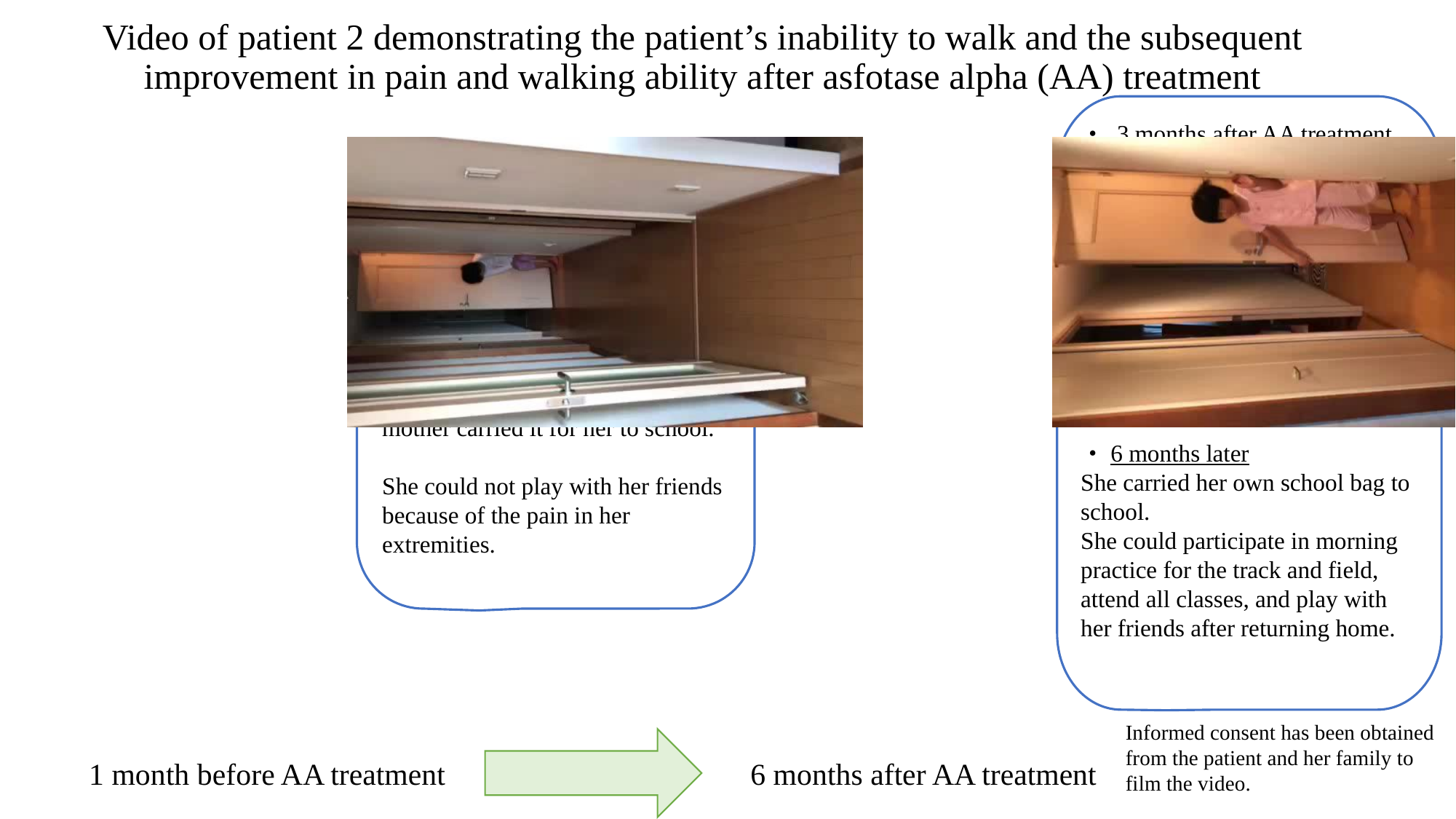

# Video of patient 2 demonstrating the patient’s inability to walk and the subsequent improvement in pain and walking ability after asfotase alpha (AA) treatment
・ 3 months after AA treatment introduction
The patient was able to participate in the field day without taking analgesic medication.
・4 months later
She attended school from the first to the sixth period.
・6 months later
She carried her own school bag to school.
She could participate in morning practice for the track and field, attend all classes, and play with her friends after returning home.
The pain in her lower back and thighs was so severe that she could not get up after waking up and tended to miss school.
Due to neck pain, she could not carry her school bag; therefore, her mother carried it for her to school.
She could not play with her friends because of the pain in her extremities.
Informed consent has been obtained from the patient and her family to film the video.
1 month before AA treatment 　　　　　　 6 months after AA treatment

## Slide 2
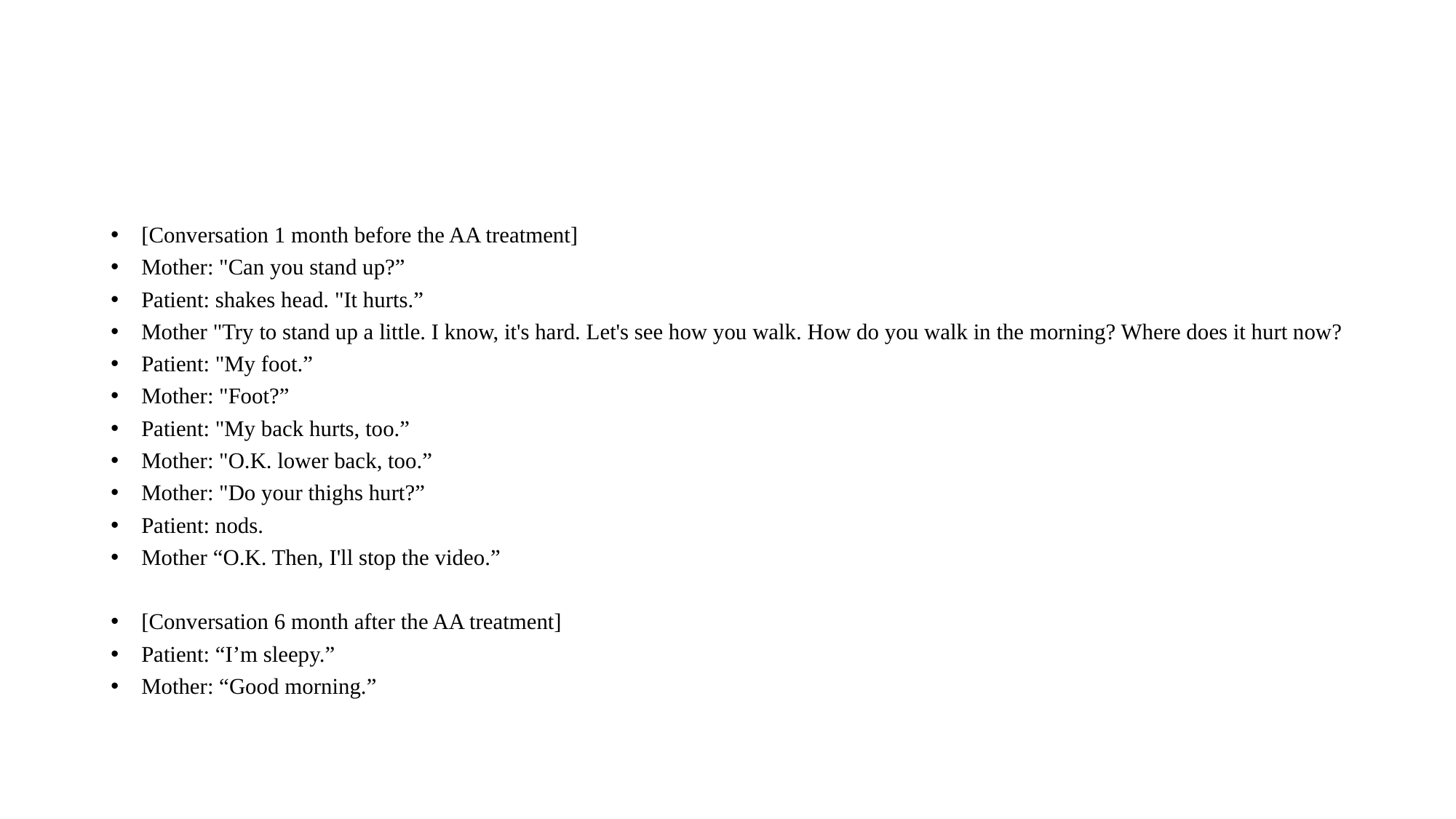

#
[Conversation 1 month before the AA treatment]
Mother: "Can you stand up?”
Patient: shakes head. "It hurts.”
Mother "Try to stand up a little. I know, it's hard. Let's see how you walk. How do you walk in the morning? Where does it hurt now?
Patient: "My foot.”
Mother: "Foot?”
Patient: "My back hurts, too.”
Mother: "O.K. lower back, too.”
Mother: "Do your thighs hurt?”
Patient: nods.
Mother “O.K. Then, I'll stop the video.”
[Conversation 6 month after the AA treatment]
Patient: “I’m sleepy.”
Mother: “Good morning.”
