# Supplementary material for: A Japanese single-center experience of the efficacy and safety of asfotase alfa in pediatric-onset hypophosphatasia
Source: Orphanet J Rare Dis. 2022 Feb 23;17:78. doi: 10.1186/s13023-022-02230-y (PMC8867653; doi:10.1186/s13023-022-02230-y)
Supplement: Supplementary file 2 — Additional file 2. Skin changes noted in each case. [file 13023_2022_2230_MOESM2_ESM.pptx]

## Slide 1
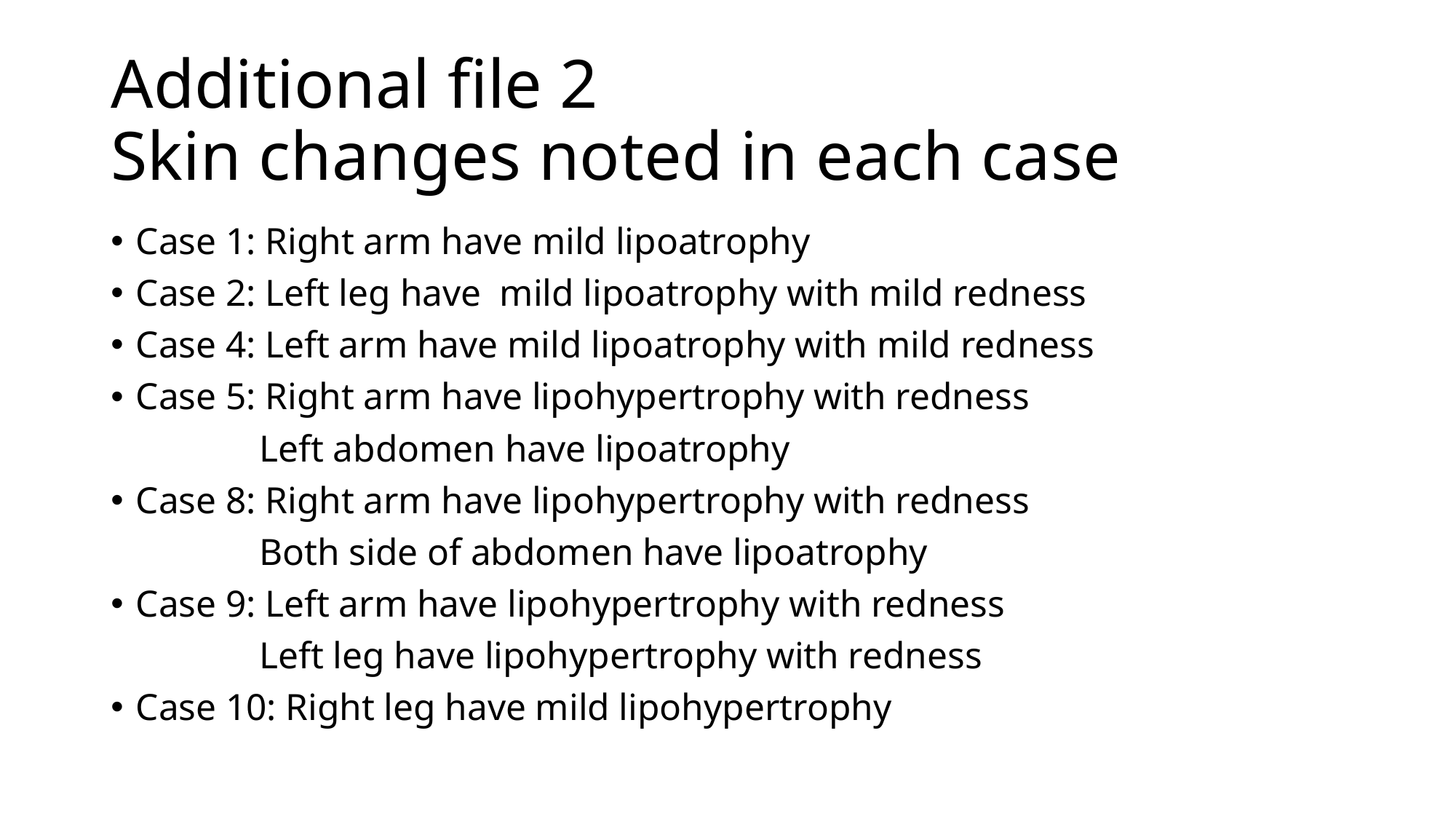

# Additional file 2 Skin changes noted in each case
Case 1: Right arm have mild lipoatrophy
Case 2: Left leg have mild lipoatrophy with mild redness
Case 4: Left arm have mild lipoatrophy with mild redness
Case 5: Right arm have lipohypertrophy with redness
 Left abdomen have lipoatrophy
Case 8: Right arm have lipohypertrophy with redness
 Both side of abdomen have lipoatrophy
Case 9: Left arm have lipohypertrophy with redness
 Left leg have lipohypertrophy with redness
Case 10: Right leg have mild lipohypertrophy

## Slide 2
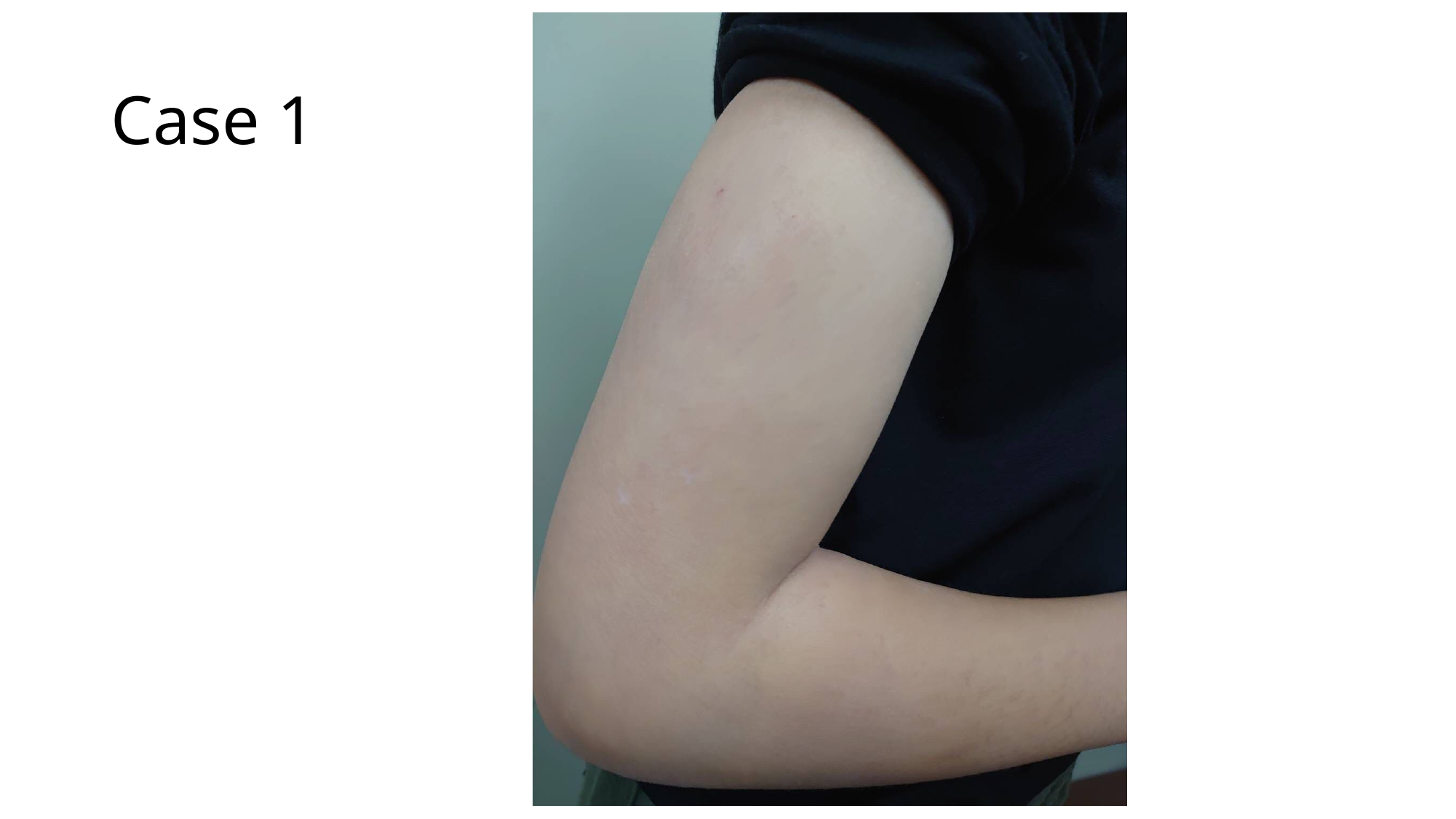

# Case 1

## Slide 3
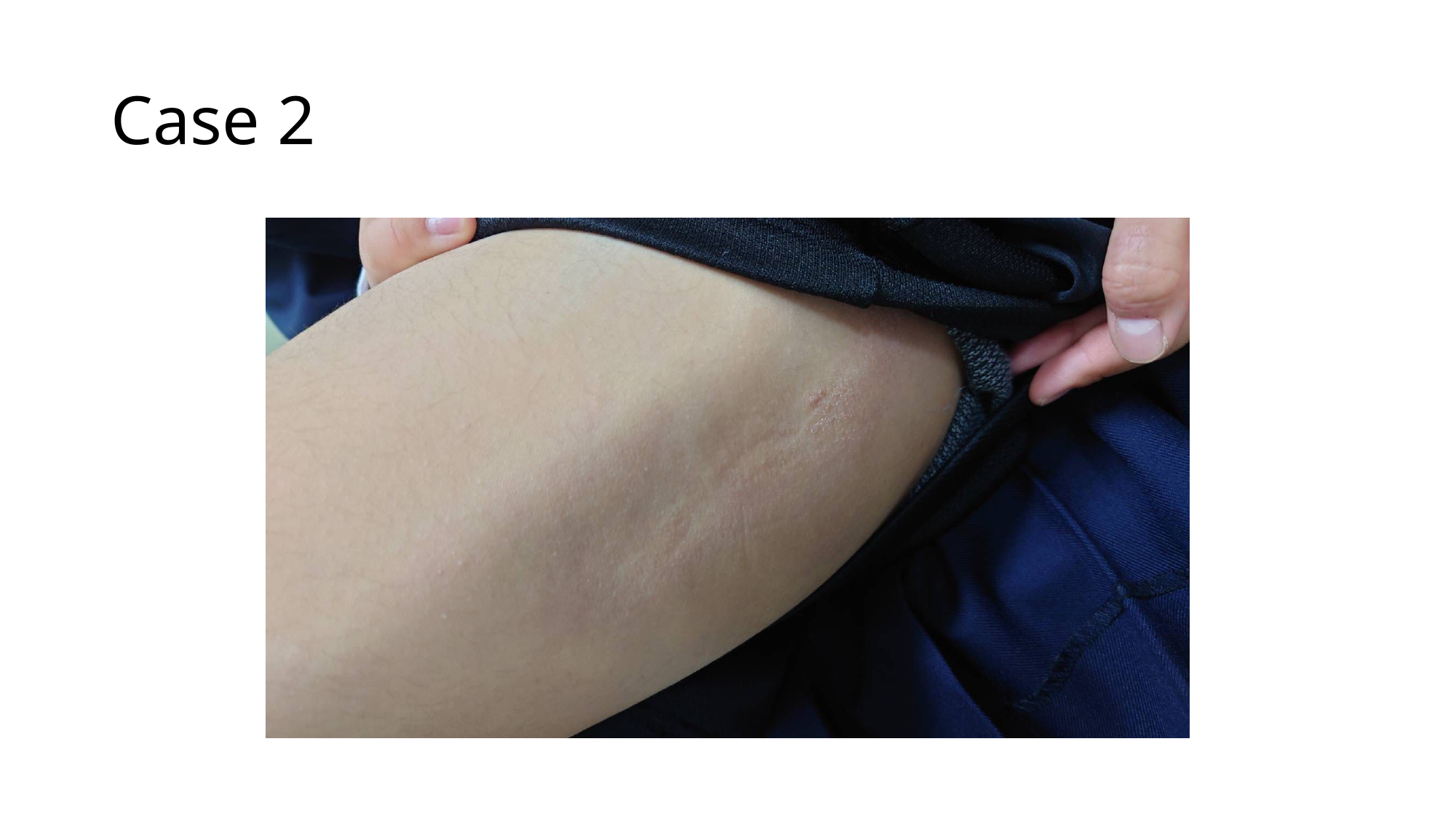

# Case 2

## Slide 4
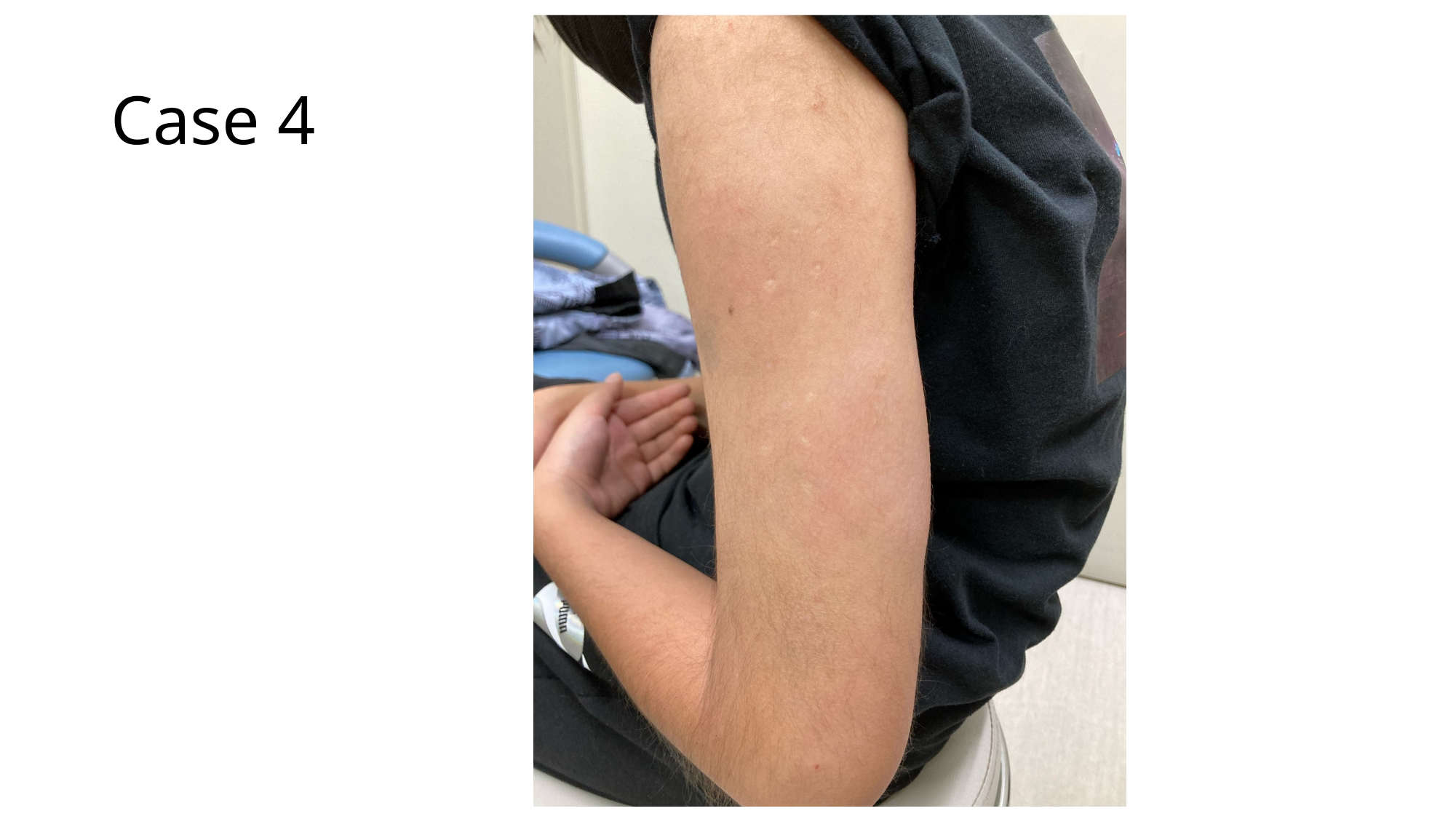

# Case 4

## Slide 5
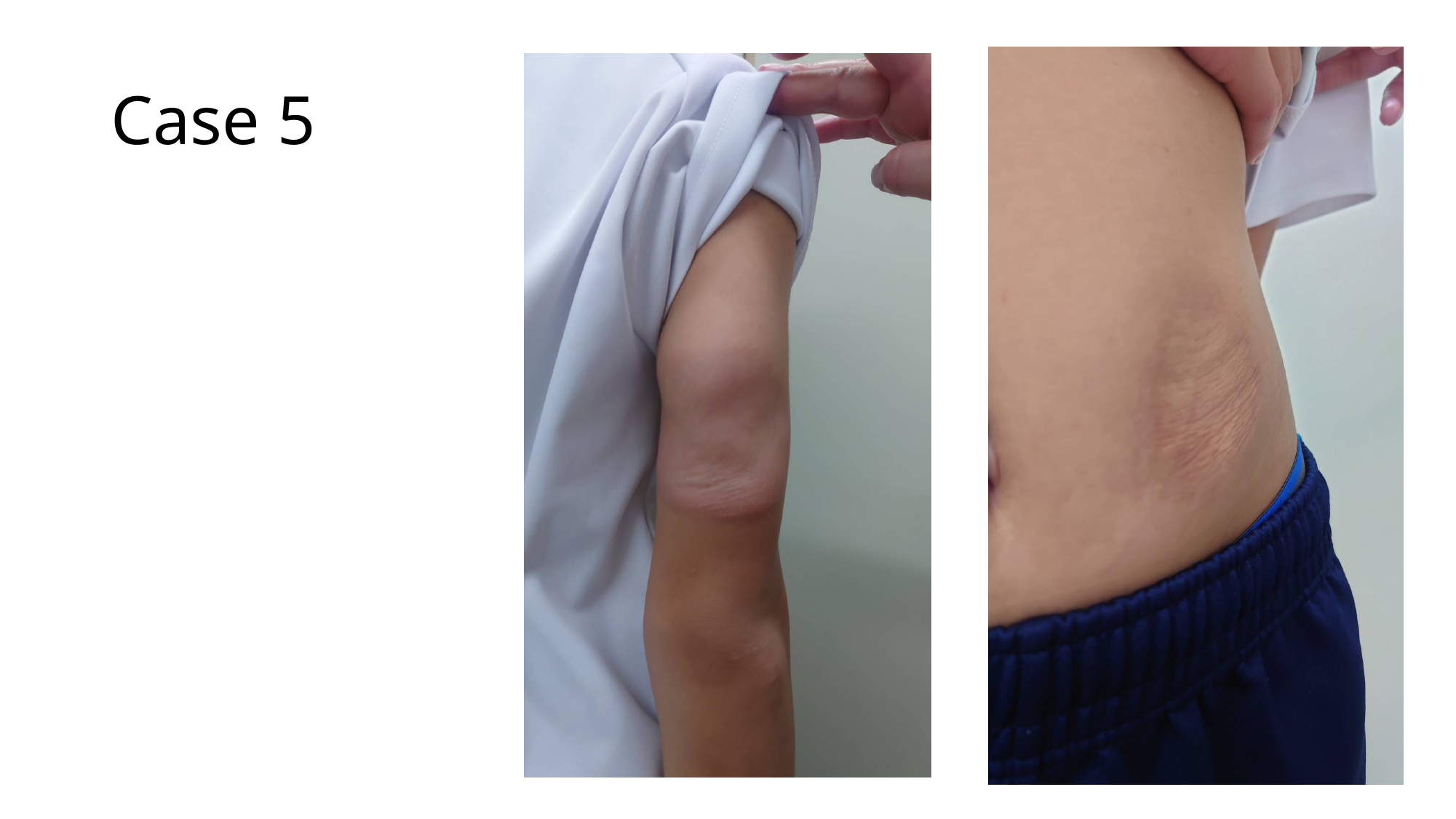

# Case 5

## Slide 6
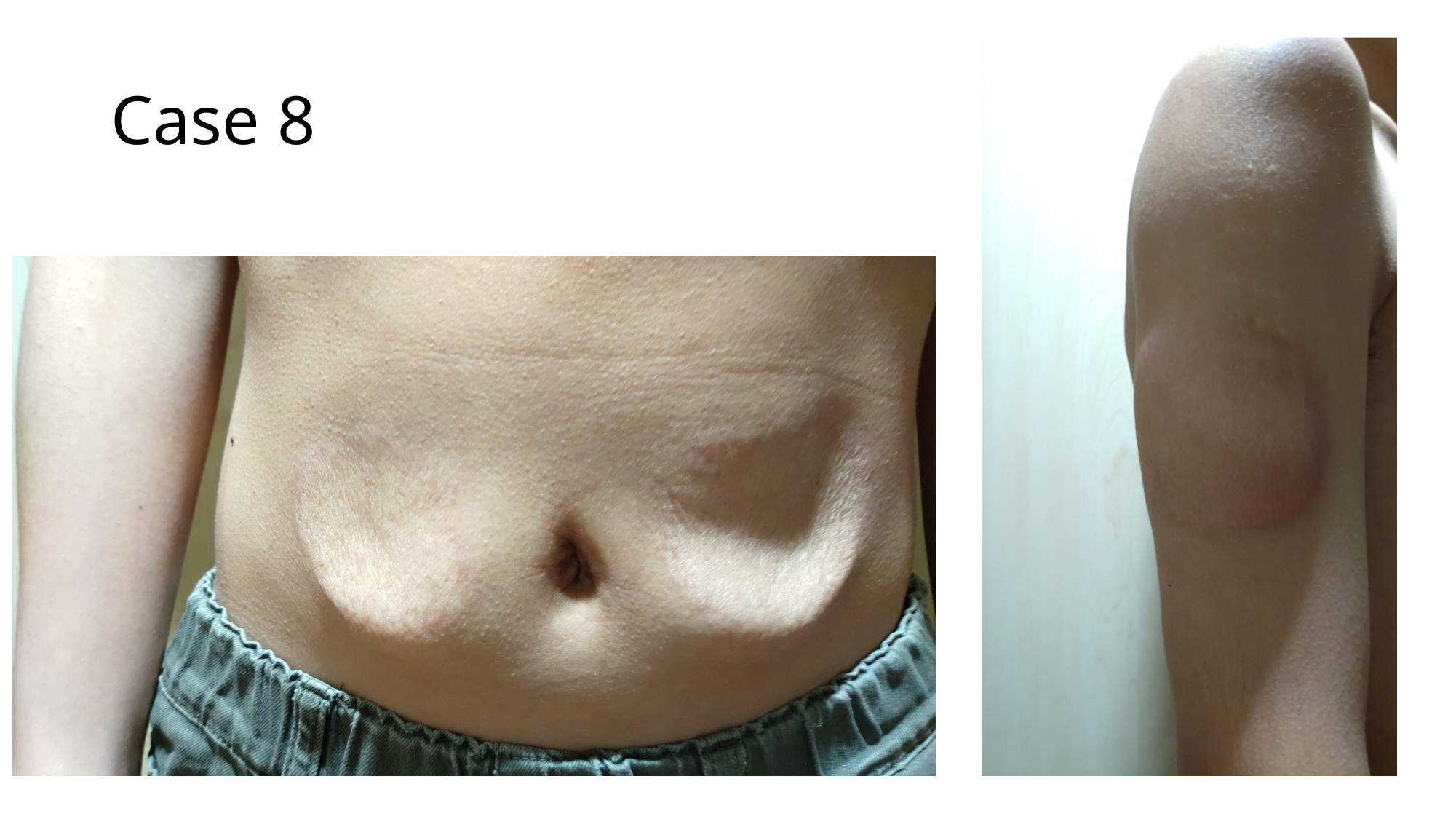

# Case 8

## Slide 7
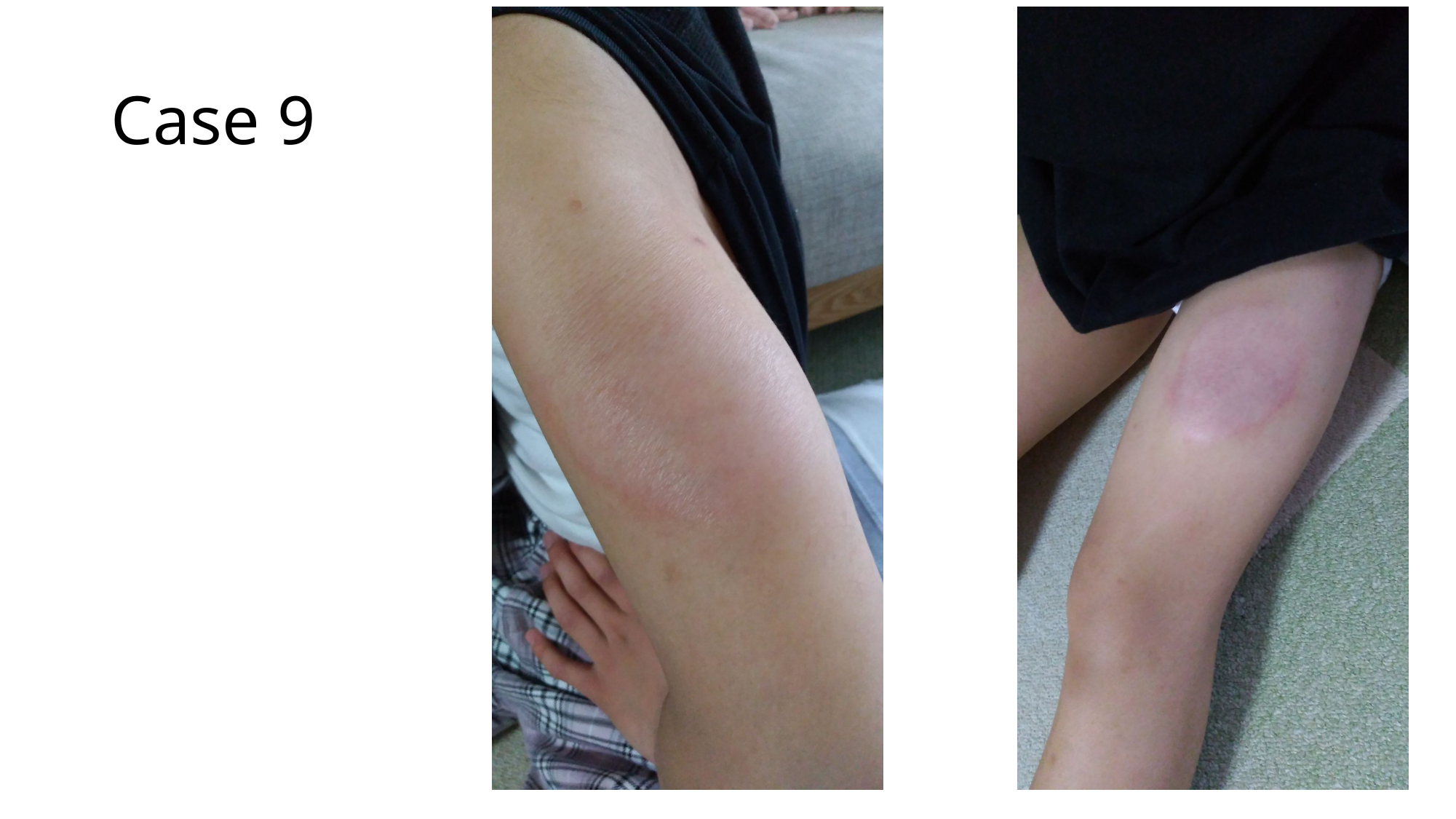

# Case 9

## Slide 8
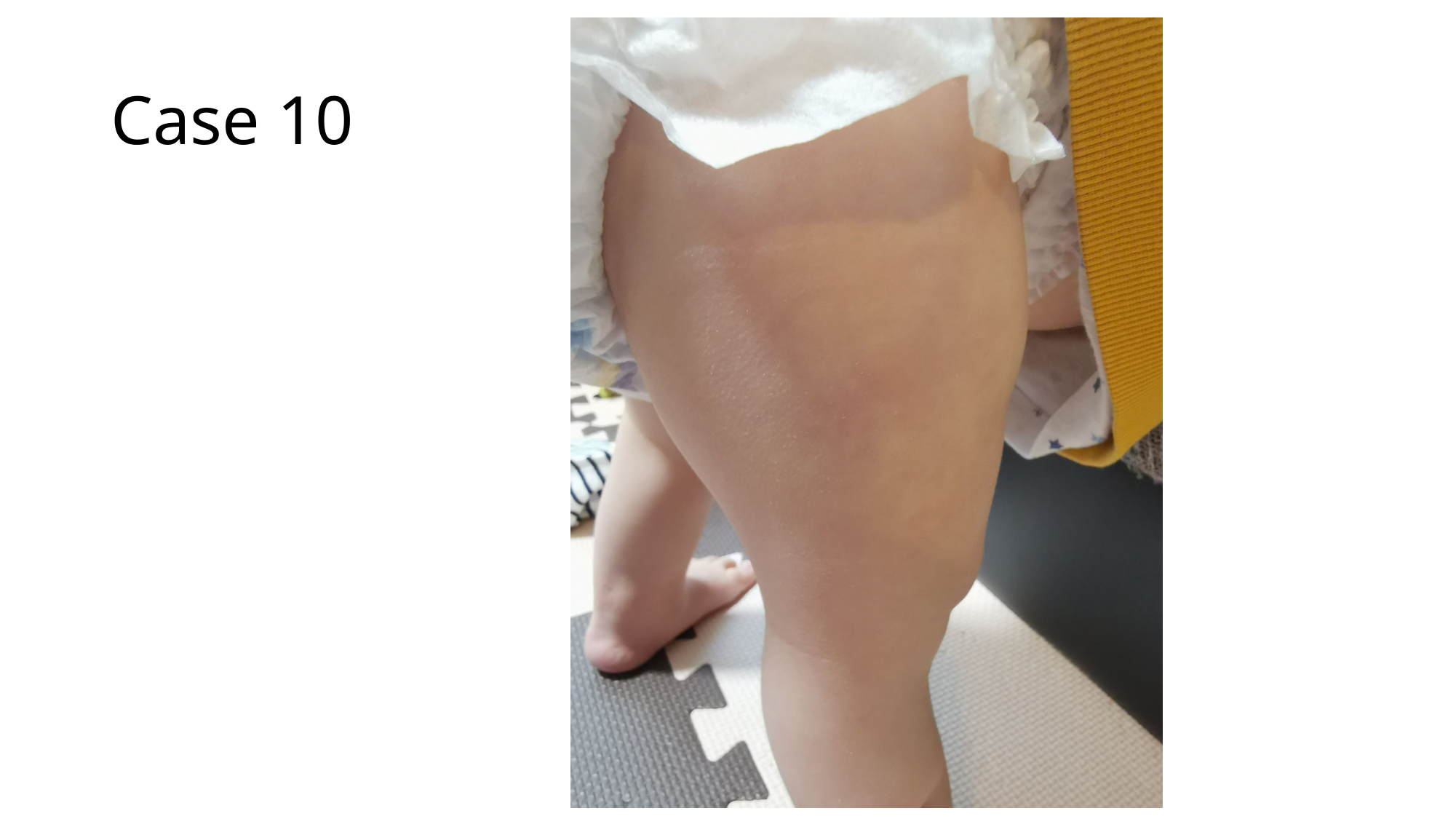

# Case 10
